# Raising the Barriers to Access to Medicines in the Developing World – The Relentless Push for Data Exclusivity

**DOI:** 10.1111/dewb.12105

**Published:** 2016-01-27

**Authors:** Lisa Diependaele, Julian Cockbain, Sigrid Sterckx

**Keywords:** data exclusivity, clinical trial data, regulatory approval, pharmaceutical industry, access to medicines, TRIPS Plus, intellectual property rights

## Abstract

Since the adoption of the WTO‐TRIPS Agreement in 1994, there has been significant controversy over the impact of pharmaceutical patent protection on the access to medicines in the developing world. In addition to the market exclusivity provided by patents, the pharmaceutical industry has also sought to further extend their monopolies by advocating the need for additional ‘regulatory’ protection for new medicines, known as data exclusivity.

Data exclusivity limits the use of clinical trial data that need to be submitted to the regulatory authorities before a new drug can enter the market. For a specified period, generic competitors cannot apply for regulatory approval for equivalent drugs relying on the originator's data. As a consequence, data exclusivity lengthens the monopoly for the original drug, impairing the availability of generic drugs.

This article illustrates how the pharmaceutical industry has convinced the US and the EU to impose data exclusivity on their trade partners, many of them developing countries. The key arguments formulated by the pharmaceutical industry in favor of adopting data exclusivity and their underlying ethical assumptions are described in this article, analyzed, and found to be unconvincing. Contrary to industry's arguments, it is unlikely that data exclusivity will promote innovation, especially in developing countries. Moreover, the industry's appeal to a property rights claim over clinical test data and the idea that data exclusivity can prevent the generic competitors from ‘free‐riding’ encounters some important problems: Neither legitimize excluding all others.

## Introduction

[T]he expansion of data exclusivity provisions has become one of the main ways of extending market protection and blocking generic competition. Data exclusivity is seen now as the principal means of extending market protection for new indications, pharmaceutical forms and other variations, especially where these are not innovative enough to gain patent protection.

(Greg Perry, European Generic Medicines Association)^1^

For products which require costly regulatory approval before they can be brought to the market, for example pharmaceuticals and agrochemicals, the ‘originators’ have traditionally sought some form of temporary monopoly, a market exclusivity, to enable them to recoup their research and development costs and to make a profit. Such a monopoly, in the paradigm case, is provided by patents. Although still widely debated, the patent‐eligibility of such products was mandated by Art. 27(1) of the *World Trade Organization – Trade Related Aspects of Intellectual Property Rights (TRIPS) Agreement*, which binds almost all countries of the world.

However the term of a patent is typically 20 years from application, and the period for which the patent is in force and the originator's product may be sold without competition can be eroded by the time required to have a patent granted and obtain regulatory approval. In many cases, at least half of the patent term may have expired before the product reaches the market. The same is not true for many inventions outside the fields of medicine and agrochemistry. Accordingly, there has been pressure to extend the period of market exclusivity for medical and agrochemical inventions.

Extended, or even *de novo*, market exclusivity has taken many forms beyond simply patent term extension. In this article we will focus on one relatively new form of (extended) market exclusivity which has grown immensely in importance since TRIPS: ‘data exclusivity’.

Data exclusivity concerns the data that the originator must submit to regulatory authorities to demonstrate the safety and efficacy of its product in order to obtain marketing approval. More particularly it concerns the extent to which a generic competitor, a ‘follower’, may rely on the originator's data in its own application for marketing approval.

Traditionally, generic ‘followers’ must only demonstrate that their drugs are bioequivalent to the original drug, and thus equally safe and effective. As a result, the follower's market entry indirectly relies on the clinical trial data already provided by the originator. The goal of data exclusivity provisions, simply put, is to delay followers from relying on the originator's data in their own applications for marketing approval for identical or similar products. During the period of data exclusivity, generic competitors are not allowed to rely on the originator's marketing approval and must either accept postponement of regulatory approval or generate equivalent clinical data.

In effect, data exclusivity provides the originator with temporary exclusive user rights to the data. Consequently, if the period of data exclusivity extends beyond the term of patent protection, data exclusivity ensures a lengthened *de facto* market exclusivity for the original product. It is a form of ‘intellectual property’ protection which, unlike patents, does not have to be applied for at an early stage of product development, and which, again unlike patents, cannot be challenged.

Besides providing market exclusivity beyond patent expiry, data exclusivity also confers market exclusivity for non‐patentable, non‐innovative drugs. Even if the originator's drug was not protected by a patent, data exclusivity can effectively prevent generic followers from entering the market. Moreover, data exclusivity allows originators to obtain market exclusivity in countries for which they did not apply for patents. Since the pharmaceutical industry's patent filing strategies in the early stages of research and development routinely omit filings in or for (most) developing countries, the effect of adopting data exclusivity can be most egregious in the developing countries.^2^

The granting of temporary exclusive user rights to data is a highly remarkable development since, traditionally, data, information, knowledge, have not been considered capable of being property which can be owned or in respect of which a company can have exclusive user rights. It has long been the case that the form in which data is presented, for example the word‐string that makes up this article, can be property protectable by copyright, but not the data itself.

Another important development is that much clinical trial data will in future have to be made publicly available. Hence, unless the originator has some exclusive user rights, there is a possibility that publicly available data might be used by a follower to support an application for regulatory approval, thereby allowing the follower to enter the market in a country where the originator has no patent or where its patent has expired or been revoked.

Encouraged by the pharmaceutical industry, both the US and the European Union (EU) seek to impose data exclusivity provisions on developing countries that go beyond the requirements of TRIPS (‘TRIPS‐Plus’ provisions).^3^ Faced with the enduring lack of access to affordable medicines, it is necessary to evaluate all policies that could influence the development and prices of drugs. This article aims to assess the legitimacy of the pharmaceutical industry's demand for data exclusivity.

First, we will describe the current status of data exclusivity provisions in US and EU law and at the international level (TRIPS). Next, we will explain the involvement of industry in pushing for ‘TRIPS‐Plus’ levels of data exclusivity, and provide examples of how Free Trade Agreements (FTAs) negotiated by the US and the EU extend beyond the provisions of TRIPS. Finally, we will set out and assess the major arguments advanced in favour of data exclusivity: (1) data exclusivity is an essential tool to promote innovation; (2) data exclusivity is a legitimate means to protect industry's property rights in clinical test data; and (3) ‘free‐riding’ by the generic industry needs to be avoided. We will conclude that these arguments are not convincing.

## The Enactment Of Data Exclusivity

While the US and the EU have had a comprehensive legal framework for data exclusivity for three decades, international standards are more recent and more controversial. TRIPS is an important milestone, but it does not mandate data exclusivity. More recent US and EU FTAs, however, have introduced stringent data exclusivity obligations for several developing countries.

### Data exclusivity in the US

The concept of data exclusivity originated in the US. In 1984, the *Drug Competition and Patent Term Restoration Act* (Hatch‐Waxman) introduced the ‘Abbreviated New Drug Application’ (ANDA) for generic drugs, allowing regulatory approval to be based on evidence that a generic drug is bioequivalent to the original. To compensate, the Act introduced a period of five years of data exclusivity.^4^ Consequently, for five years, a follower cannot obtain marketing approval by relying on the originator's data. A generic competitor needs to submit independently generated clinical data or delay its application.

Besides five years of data exclusivity for all new chemical entities, additional protection was granted for specific categories of drugs and clinical data. Where a new drug is recognized as an ‘orphan drug’ – for the treatment of rare conditions – a period of seven years of data exclusivity applies. For data that support changes to products already on the market (such as new indications, new dosages and new delivery methods), ‘clinical investigation exclusivity’ limits market authorizations for three years. The submission of data to support the paediatric use of an existing drug lengthens the period of data exclusivity by six months.

### Data exclusivity in the EU

Following the US, the EU adopted a regulation in 1987, mandating a period of data exclusivity of at least six years. In 2004, the EU extended this to ten years. This delay can be extended for another year ‘if, during the first eight years of those ten years, the [originator] obtains an authorisation for one or more new therapeutic indications which … bring a significant clinical benefit in comparison with existing therapies.’^5^ As in the US, the EU has introduced a separate regime of ten years of data exclusivity for orphan drugs.

### The TRIPS Agreement: the protection of undisclosed data against unfair commercial use

It is argued that TRIPS set the first international standard regarding data exclusivity. However, TRIPS does not impose such an obligation – Art. 39(3) merely requires the protection of undisclosed data against ‘unfair commercial use’:

Members, when requiring … the submission of undisclosed test or other data, the origination of which involves a considerable effort, shall protect such data against unfair commercial use.

TRIPS does not define ‘unfair commercial use’. It is hard to see how the ‘reliance’ of a regulatory authority on the originator's data could constitute a ‘commercial use’. At one extreme, a follower may submit the originator's data – at the other it may just ask the regulator to rely on that data. In the latter case, the regulator may refer to the originator's data or it may rely on the fact that sufficient data has been presented to it or to another country's regulator. It is only in the first case that it can clearly be said that there is ‘commercial use’ of the data.^6^ Moreover, the Paris Convention – to which the first paragraph of Art. 39 TRIPS refers – defines ‘unfair competition’ as acts ‘contrary to honest practices in industrial or commercial matters’ such as false allegations and misleading.^7^ The granting of exclusive rights is not mentioned at all.

### Data exclusivity in bilateral agreements with the US and the EU

While the US and the pharmaceutical industry continue to argue that TRIPS does require the adoption of data exclusivity,^8^ they have also sought more specific and stringent standards in bilateral and regional agreements. Since TRIPS, both the US and the EU have consistently urged their trade partners to undertake increased protection of all intellectual property rights in bilateral and regional FTAs.^9^ Especially regarding regulatory protection – including data exclusivity and patent linkage^10^ – these TRIPS‐Plus agreements have significantly raised the standards.

In 1994, the North American Free Trade Agreement (NAFTA) between the US, Canada and Mexico, was the first supranational agreement to include a specific obligation to adopt data exclusivity. In addition to an obligation to protect clinical test data against disclosure and unfair commercial use, Art. 1711(6) NAFTA specifies that, without permission, no one may rely on these data in support of an application for marketing approval for ‘a reasonable period of time, normally not less than five years.’^11^

In contrast, more recent agreements employ a stricter wording. The US‐Chile FTA (2004) was the first to require ‘a period of at least five years from the date of approval for a pharmaceutical product and ten years from the date of approval for an agricultural chemical product’ (Art. 17(10)). This wording has been standard ever since.

Several other US FTAs have raised the bar for data exclusivity further by expanding the scope of the obligations. Whereas some early agreements limited data exclusivity to ‘new chemical entities’ and for clinical data that involved ‘considerable effort’, Art. 16(8) of the US‐Singapore FTA (2004) requires data exclusivity for all regulatory approvals. Moreover, since 2005, many US bilateral agreements introduced a separate regime of data exclusivity for new clinical information, bringing standards even closer to US regulations.^12^

Some FTAs also require data exclusivity even when the regulatory authority does not require the submission of data, but instead relies on regulatory approval in another country. For example, Art. 15(10) of the Dominican Republic‐Central America Free Trade Agreement (2004; DR‐CAFTA) forbids the marketing of pharmaceutical and agricultural chemical products ‘on the basis of (1) evidence of prior marketing approval in the other territory, or (2) information concerning safety or efficacy that was previously submitted to obtain marketing approval in the other territory, for at least five years for pharmaceutical products and ten years for agricultural chemical products…’. As a consequence, if a drug is not marketed in a country by the originator, a follower cannot enter the market either, unless it independently generates the data. Moreover, most agreements specify that the term of data exclusivity is to be counted from the date of the initial approval in the approving country, which can be significantly later than the initial approval in the US.^13^

Since the revision of its initial FTA with Peru in 2007, waiving the obligation to grant data exclusivity when approval is based on prior approval in another country,^14^ more recent US agreements with Panama (2011; Art. 15(10)) and Colombia (2011; Art. 16(10)) also contain slightly ‘softened’ standards: the application of data exclusivity is limited to the approval of ‘new chemical entities’, for clinical data that involved ‘considerable effort’ and for a ‘reasonable period’, normally five years.

The EU has also tabled proposals regarding data exclusivity as a TRIPS‐Plus requirement during its trade negotiations, although less frequently than the US.^15^ In 2012, the EU concluded the EU‐Peru‐Colombia FTA, of which Art. 231(4)(a) requires five years of data exclusivity for pharmaceuticals and ten years for chemical agricultural products.^16^ Importantly, this FTA foresees the possibility to regulate ‘exceptions for reasons of public interest, situations of national emergency or extreme emergency’, indicating the possibility of granting market access for generic drugs to address health emergencies. The EU‐South Korea FTA (2010; Art. 10(36)) also specifies a period of five years of data exclusivity, and the EU‐Canada agreement forbids the marketing approval of generics relying on originator's data for eight years. (Chapter 22, Art. 10).

While the total number of countries currently bound to enact data exclusivity regulations might seem limited, the impact of these TRIPS‐Plus requirements should not be underestimated. The incorporation of data exclusivity provisions in FTAs has become the new standard. For example, the recently concluded Trans Pacific Partnership (TPP) provides for an elaborate data exclusivity regime. In addition to five years of data exclusivity for new chemical entities and three years for new clinical information, the TPP is the first treaty providing a specific data exclusivity regime for biologics, mandating eight years of data exclusivity, or five years combined with additional measures.^17^ If the TPP is ratified, a total of 12 countries, representing 40% of the global GDP, will be required to incorporate these measures.^18^

## The Role of the Business Communities in Securing Data Exclusivity

It is clear from the documents regarding the negotiation of TRIPS that the development of international intellectual property law has been significantly influenced by business communities. Both before and during the TRIPS negotiations, the United States Trade Representative (USTR), directly influenced by business interest groups, vigorously pursued the inclusion of substantial minimum standards for the protection and enforcement of intellectual property rights in the GATT, the precursor to the WTO.^19^ Especially in the first years of the Uruguay Round negotiations, significant efforts had to be made by the negotiating countries’ trade administrations to gather the necessary information and expertise, offering business lobby groups the opportunity to fill some of the space.^20^

Regarding data exclusivity, similar dynamics have occurred. Both in the US and the EU, business interest groups actively lobbied to secure data exclusivity. Although clinical data could be protected as trade secrets in the EU and followers could not enter the market without regulatory approval, member states’ regulatory authorities were more permissive about the reliance on originator's data to grant regulatory approval to generics. After data exclusivity was introduced in the US in 1984, the European pharmaceutical industry actively lobbied to obtain similar protection in the EU. They managed to persuade the European authorities that this would boost pharmaceutical research and innovation in Europe. They claimed that data protection in the US gave American counterparts a competitive advantage and that, in order to gain competitive edge, the EU should adopt longer data exclusivity periods than the US.^21^ The European Federation of Pharmaceutical Industries and Associations (EFPIA) requested a harmonized period of data exclusivity in the EU of ten years. Throughout the preparation of the ‘pharmaceutical review’ – a broad package of legislative proposals aimed at harmonizing the regulatory framework for pharmaceutical development – EFPIA managed to position itself as an indispensable expert to both the European Commission and the European Parliament.^22^

Multinational pharmaceutical companies continue to play a similar instrumental role in the propagation of global intellectual property rights.^23^ Regarding data exclusivity, initial efforts focused on ‘compliance’ with Art. 39 TRIPS. For example, in 2000, the International Federation of Pharmaceutical Manufacturers & Associations (IFPMA) issued a report, describing clinical data as ‘proprietary registration data’ and data exclusivity as an ‘independent intellectual property right’ that had to be protected in order to be TRIPS‐compliant.^24^ Although this is highly questionable,^25^ the USTR adopted the same approach:

the TRIPS Agreement recognizes that the original applicant should be entitled to a period of exclusivity during which second‐comers may not rely on the data that the innovative company has created to obtain approval for their copies of the product. During this period of exclusive use, the data cannot be relied upon by regulatory officials to approve similar products.^26^

Ever since, business interest groups and pharmaceutical companies have continuously urged the USTR to demand third countries to provide data exclusivity.^27^ Pharmaceutical Research and Manufacturers of America (PhRMA) – a key industry group – even suggests that the US should take ‘aggressive action’ – trade sanctions and international dispute settlement procedures – to remedy these alleged intellectual property violations.^28^

The USTR is at risk of ‘regulatory capture’, of being dominated ‘by private interest groups that the agency is responsible for regulating.’^29^ Therefore, it is critical to examine how private interest representation is organized. The USTR – advised by the Industry Trade Policy Advisory Committee on Intellectual Property Rights (ITAC 15), consisting of representatives of key industries^30^ – is exempt from federal regulatory mechanisms to ensure a balanced representation of interests and public access to information, leaving a giant loophole. In practice, ITAC 15 does not consult other industries, public interest groups or academic experts.^31^ Moreover, the USTR is not even required to make its communications with industry advisers public.^32^

An important tool in the formulation and implementation of US external trade policies are the ‘Special 301 Reports’. The USTR lists countries on ‘watch lists’ if they fail to adequately protect US commercial interests. In the last decade, ‘sufficient protection’ of clinical test data has become an important parameter in this context. For example, the 2015 report highlights ‘serious obstacles’ to the effective protection of pharmaceutical test data as important issues for 18 countries, all developing countries and emerging economies.^33^

Even though many NGO's and non‐profit organizations such as Oxfam, Public Citizen and Knowledge Economy International have urged the USTR to reconsider its position on data exclusivity,^34^ their impact seems limited. The policy formulation process – which closely involves industry representatives but remains shielded from public scrutiny – as well as the policy outcomes – which clearly favour the industry's requests – suggest that the USTR is successfully influenced by the pharmaceutical industry.

## The Arguments Invoked for Data Exclusivity

The arguments, invoked to legitimize the industry's pursuit of increased protection, can roughly be divided into three. First, data exclusivity is said to be an essential policy tool to *promote innovation*. According to the second argument, data exclusivity is a legitimate measure to protect *property rights* in clinical trial data. The third argument is one of *‘justice’* – that followers should not be free to use information generated by originators since ‘free‐riding’ is unfair and thus wrong.

The first, consequentialist, line of argument is that data exclusivity is necessary to allow pharmaceutical companies to recoup the costs of conducting clinical trials. Clinical trials require significant investment, and because there might be little or no patent protection left at the time of marketing, some additional years of data exclusivity are said to be essential financial incentives. Thus, according to the proponents, data exclusivity ‘helps to ensure a limited period during which an adequate return on … investment can be made.’^35^ Furthermore, it is claimed that incentivizing clinical trials will encourage the development and marketing of non‐innovative drugs.^36^

If a country provides this incentive, R&D investments and innovation are promised to increase. Especially in a global pharmaceutical market, according to IFPMA, it would be unwise for countries not to adopt data exclusivity as:

countries which offer data exclusivity are encouraging businesses to move their product, investment and potential manufacturing to their markets earlier. If other companies could immediately use these data to obtain their own marketing authorization … there would be less incentive for the innovator to invest ….^37^

PhRMA also seeks to legitimize its demand for the global recognition of data exclusivity by pointing out that not all countries grant patent protection for new biological drugs, which are more difficult and costly to produce than traditional pharmaceuticals. ‘In these countries, data protection may provide one of the few incentives for regionally specific innovation and may provide an important incentive to launch new innovative products in the country.’^38^ For example, BIO – the Biotechnology Industry Organization – advocated the adoption of a twelve year data exclusivity period for biologicals in the Trans‐Pacific Partnership (TPP).^39^

The second line of argument is that data exclusivity is a legitimate measure to protect the property rights of the pharmaceutical industry over the clinical trial data they generate. Essentially, because the pharmaceutical industry financed and generated the clinical data, they own the data: ‘The results obtained are as much the property of the company that produced them as is the plant used to manufacture the product.’^40^ Indeed, pharmaceutical industry associations frequently employ terms such as ‘proprietary test data’.^41^

Third, data exclusivity is often described by the pharmaceutical industry as a necessary means, in addition to patent protection, to prevent the generic industry from ‘free‐riding’.^42^ Since the originator needs to make a significant financial investment to generate the clinical data, direct or indirect reliance on the original clinical data by others is seen as an unjust competitive advantage, ‘unjust enrichment’ or ‘unfair commercial use’, even in the absence of fraud or dishonesty.^43^

Finally, another (mostly unmentioned) reason for the pharmaceutical industry to strive for the adoption of data exclusivity is the increased tendency towards clinical trial data transparency. After extensive lobbying by public interest groups, the new EU clinical trials legislation, which will enter into force by May 2016, will require the registration of all clinical trials in an EU database, making clinical trial results publicly available.^44^ A similar trend can be witnessed in the US.^45^ From the perspective of the pharmaceutical industry, this is an increasingly worrying trend for, if the results of clinical trials become publicly available, clinical trial data are no longer ‘undisclosed data’, and, absent data exclusivity, can thus be used by followers in support of their applications for marketing approval. Clearly, the continuous push by the pharmaceutical industry for stringent data exclusivity standards seeks to neutralise the effects of this trend of increasing transparency regarding clinical trial data.

## Assessing the Arguments

In order to assess the legitimacy of the pharmaceutical industry's quest for increased protection of clinical data, we will take a closer look at the arguments mentioned in the previous Section. Considering the enduring lack of availability and affordability of essential medicines, we will pay particular attention to the potential impact of data exclusivity in developing countries.

### The innovation argument

#### The cost of drug development

The argument that data exclusivity is necessary to incentivize innovation is based on particular claims regarding the cost of pharmaceutical research and development. However, the actual costs of drug development are highly debated. Estimates vary significantly, but most figures cannot be independently verified because the industry systematically refuses to disclose the underlying data for independent review.^46^ Industry associations usually refer to the Tufts Center for the Study of Drug Development (CSDD) – an institute established as a result of a conference held at the Chicago School of Economics with funding from the pharmaceutical industry.^47^ The CSDD's most recent estimates report drug development costs of up to 2.6 billion USD.^48^

Obviously, it is in industry's interests to portray R&D costs as being as high as possible, and thus only to report aggregate data which include failures and the cost of capital, and without crediting government subsidies. Consequently, according to some commentators, the actual costs of drug development may be as low as a quarter of the reported costs.^49^ Nevertheless, it is clear that drug R&D requires significant investment, and thus that originators need an opportunity to at least recoup their expenses. However, is data exclusivity necessary to achieve this?

The industry claims that costs have increased significantly, particularly due to the costs of clinical development. However, the costs looks meagre compared to total revenues: PhRMA itself reports an increase of 34.2 billion USD in costs between 1995 and 2010 but a six‐fold increase in revenues of 200.4 billion USD for the same period.^50^

Furthermore, a look at the top 100 US drug sales for 2013 shows that 55 ‘blockbusters’ each generated over 1 billion USD.^51^ Even if a drug would only have a couple of years of effective patent protection, this should suffice to cover the costs. Overall, the pharmaceutical industry remains hugely profitable. For 2013, the top 20 pharmaceutical companies each reported profit margins of 22.3‐59.7%, and incomes of 2.5‐15.9 billion USD.^52^

Clearly, these figures question the necessity of providing data exclusivity to enable recoupment of drug development costs. At the very least, requiring developing countries to implement data exclusivity is totally unnecessary.

#### Data exclusivity and pharmaceutical innovation

Data exclusivity can increase the profits of the pharmaceutical industry. Industry claims that, by offering this financial incentive, data exclusivity also increases innovation. Unfortunately, hardly any empirical research is available. However, because data exclusivity *de facto* confers or lengthens market exclusivity, it must have similar effects to those of patents, hence findings regarding the effects of patent protection on innovation can reveal important trends.

Intense debate exists among economists, policy experts and industry, as to whether or not (strengthening) the patent system stimulates innovation. Much research is based on theoretical economic models, assuming that investments in R&D will automatically increase when the expected financial incentives adequately compensate the risks and costs of R&D.^53^ However, this ‘Schumpeterian model’ of innovation has its flaws. Indeed, there seems to be a point beyond which increased protection will no longer benefit innovation.^54^ Moreover, strong patent protection can hinder innovation, for example by delaying sequential innovations.^55^ Data exclusivity might not prevent, but instead discourage innovation, by incentivizing low‐risk investment. Especially for non‐innovative drugs, data exclusivity offers industry a lucrative opportunity since the development of such drugs costs significantly less and, despite the lack of patent protection, a market monopoly for several years can be obtained through data exclusivity.

The assumption that increased protection will automatically encourage innovation is thus questionable. Most empirical data show a much more nuanced picture. Key to a correct interpretation is what exactly is measured, and in which countries. Cross‐country data indicate that the positive correlation of patents with innovation – measured by R&D investments and patent applications – is only consistently positive in developed and higher‐income emerging economies. For developing countries, empirical results do not systematically indicate a positive correlation.^56^ Moreover, when compared to the global increase of patent applications, applications by domestic applicants have declined.^57^ Clearly, the argument that adopting data exclusivity could generate an advantage for domestic industry is false. Foreign companies equally enjoy the benefits of data exclusivity.^58^

It is often assumed that a rise in patent applications by foreign firms in a country that increases patent protection will lead to an increased transfer of technology and innovation. Yet the positive effects of patent protection on technology transfer also seem limited to large‐ to middle‐income countries.^59^ Equally, the effects of increased patent protection on R&D investments by foreign firms mostly occur in developed and emerging economies.^60^ In developing countries, positive effects are scarce.^61^ In Jordan, for example, the implementation of ‘TRIPS Plus’ levels of patent protection and adoption of a data exclusivity regime following the conclusion of an FTA with the US, did not result in any additional foreign investment in pharmaceutical manufacturing or R&D, nor did it encourage domestic innovation.^62^

In sum, there is little evidence that increasing protection has had a positive impact on economic development and innovation in countries in the developing world, which remain net importers of technology.^63^ In addition to this problem, there is no systematic evidence of a causal relationship between increased patent protection and innovation.^64^ Although many studies find a positive correlation between strong patent protection and innovation, this can mostly be explained by other factors such as educational attainment and economic freedom.^65^ As most studies recognize, the positive effects of intellectual property rights mainly depend on a country's innovative ability.^66^

The argument that adopting data exclusivity would support the development of drugs for the diseases that mainly affect poorer populations in developing countries, is also feeble. The current business model relies on wealthy markets and public and private insurers paying the bills. In the absence of solvent ‘consumers’, market exclusivity may not provide a sufficient incentive for R&D investment.^67^ Interestingly, empirical data also indicate that the acceptance of stronger patent protection by its foreign trade partners does not have a significant impact on innovation in the US:

It probably implies that the patent‐protected US market is sufficiently large for innovators to recoup the costs of R&D investments and further strengthening IPR protection by individual foreign countries merely adds pure rent to the proceeds that US innovators earn.^68^

While innovation can be a legitimate goal, market exclusivity may not be the best way to encourage it, especially in developing countries. In the best case, data exclusivity can encourage some innovation and benefit some actors, but not necessarily the ‘innovation’ that patients need. Data exclusivity does not compensate the financial ‘risk’ of R&D, as the highest costs come at a time when the risks of failure are lowest and the time to market short.^69^

Hence, the argument that data exclusivity is necessary to encourage innovation is insufficiently supported by empirical evidence. With regard to developing countries, this conclusion is even more pertinent. In many developing countries, there is no market for high‐priced pharmaceuticals. In the absence of other factors encouraging innovation, data exclusivity does not encourage innovation.

#### Data exclusivity and (affordable) access to medicines in developing countries

In many developing countries, public health institutions cannot provide essential medicines to patients. Moreover, even if essential medicines are available, they remain unaffordable for billions of people. Especially original brand medicines are ‘priced out of reach’.^70^ Although many factors can increase the accessibility and affordability of essential medicines, the United Nations (UN) and the World Health Organization (WHO) highly recommend that developing countries make full use of TRIPS flexibilities and facilitate the production and importation of generics.^71^

In many cases, data exclusivity will delay the availability of new generics. A recent study showed that the implementation of a data exclusivity regime in Guatemala, mandated by DR‐CAFTA, resulted in generic competition being denied entry to the Guatemalan market.^72^ In each case, the available originator drugs were priced substantially higher.^73^ Especially in those countries which, pre‐TRIPS, did not grant patents for pharmaceuticals, data exclusivity can be an efficient method to ensure market exclusivity for originator drugs and prevent generic competition in that market.

As the access to medicines in the developing world is a highly complex issue, simply not providing data exclusivity cannot by itself resolve the lack of basic healthcare infrastructure in many developing and least‐developed countries. However, for both governments and individuals, the price of medicines can be a significant financial burden. Although generics are not necessarily affordable for all, the prices of original drugs tend to be at least ten times higher.^74^ Because most developing countries rely strongly on generics, the consequences of implementing data exclusivity could be enormous.^75^

Data exclusivity also offers industry the opportunity to ‘optimize’ its global business strategy. Pharmaceutical companies do not file patent applications in all the countries where they will eventually market their products. The inclusion of data exclusivity in FTAs ensures market exclusivity without a patent. Furthermore, companies will first introduce new drugs in wealthy markets, where they expect the best commercial opportunities. Only at a later stage, are new drugs marketed in developing countries. Consequently, delaying marketing approval ‐ by means of data exclusivity ‐ can equally delay generic competition.

In sum, data exclusivity poses an additional hurdle to affordable access to medicines in developing countries. In the absence of evidence that data exclusivity supports innovation and countries’ economic development, there seems to be no legitimate ground for developing countries to adopt it, let alone strengthen it.

### The property rights argument

An entirely different argument invoked by the pharmaceutical industry is that data exclusivity is a legitimate measure to protect their property rights over the clinical trial data they generate. This gives rise to the question as to whether anyone can legitimately claim a property right to data.

Data exclusivity limits reliance on the knowledge that clinical data brings us – that a drug is safe and effective and can be allowed on the market. As mentioned earlier, knowledge is traditionally considered to be incapable of being property, in contrast to the forms in which knowledge can be presented. It is the very nature of knowledge to be a public good. When clinical data prove that a drug is safe and effective, everyone knows that equivalent drugs will be safe and effective as well.

Assuming for a moment that industry's investment in clinical trials would legitimate a property claim, why should this necessitate an unalienable exclusive user right? Having a property right does not imply an exclusive user right, especially when the interests of society as a whole are at stake. Indeed, most patent laws allow exceptions to the exclusive rights of patent holders. For example, the TRIPS Agreement maintained the possibility of issuing compulsory licences^76^ to address public health emergencies. In contrast, most data exclusivity regimes do not allow any public interest exceptions. Data exclusivity could even undermine the flexibilities allowed by TRIPS, by preventing compulsory licensed generics from obtaining marketing approval.

### The free‐riding argument

The third argument invoked by industry portrays the reliance of generic followers on originators’ clinical data as ‘free‐riding’, giving the generic industry an ‘unjust’ competitive advantage. However, this argument from ‘justice’ faces severe problems and does not imply an absolute right to exclude others, as mandated by data exclusivity.

Generally speaking, our lives as socialised humans are founded on free‐riding. In all aspects of life – economic, cultural, and scientific – people rely on earlier efforts made by others. One cannot dispute that the reliance of the generic competitor on the originator's efforts to produce clinical data constitutes an advantage. However, that does not mean the advantage is ‘unfair’ or ‘unjust’. For innovative drugs, the patent system already makes an exception to free competition to account for the originator's investment. Adding a further temporary monopoly under the guise of data exclusivity does nothing to stop free‐riding; it is merely delayed.

Moreover, even without data exclusivity, the originator's investment in clinical data is not without benefit; it provides a ticket to being the first mover on the market, entitled to make a profit until others arrive on that market.

Furthermore, whereas patents are often challenged and revoked, data exclusivity cannot be challenged. Considering that market exclusivity can bring about significant societal costs and impacts, this is unfair. Even if a generic competitor would successfully challenge a patent on a drug currently on the market, this drug could maintain its market exclusivity relying on data exclusivity. The pharmaceutical industry, on the other hand, can take full advantage of the possibility to use litigation. Even if proceedings are unsuccessful, they can delay the access of generics to the market. Consultations organized by the European Commission indicate that, between 2000 and 2007, over 700 lawsuits for patent and data exclusivity infringements were initiated by the pharmaceutical industry, but only 2% of the claims were recognized.^1^

Additionally, demanding that the generic industry duplicates clinical trials in order to avoid ‘free‐riding’ is unethical, not least because Phase I clinical trials require testing of drugs (which almost inevitably have some undesirable side‐effects) on healthy ‘volunteers’ and Phase II trials require dosage optimisation on patients for whom knowingly incorrect dosages may be detrimental, if not fatal.

Finally, even if the reliance of the generic competitor on the originator's efforts to produce clinical data would be an ‘unfair’ advantage – this does not legitimize granting exclusive rights, as is the case with data exclusivity. Fair competition could also be established by asking generic competitors to pay a contribution for their ‘use’ of the clinical data. Given the adverse consequences of excluding generic competition, this would undoubtedly be a more legitimate option. However, the mere fact that an argument from justice would not entirely preclude any system of compensation, does not mean that compensations should be paid.

## Concluding Remarks

There seem to be few, if any, reasons left to accept data exclusivity in addition to the existing patent regime. Data exclusivity poses a considerable additional risk to the affordable access to medicines in developing countries. In the absence of evidence that data exclusivity will support innovation and economic development, there is no legitimate ground for developing countries to favour such a policy. Moreover, since current levels of revenue already generate copious profit margins for the pharmaceutical industry in US and EU markets, it is inequitable and highly problematic to require developing countries to implement data exclusivity.

For developed country markets, the key question remains whether society should pay the price for extended monopolies in return for merely ‘incremental’ innovations. Even in the US and the EU, the implementation of data exclusivity, by undermining legitimate competition, seems incompatible with the long tradition of stringent competition and anti‐trust policies, which have always been vital components of the economic structure. In its current form, data exclusivity offers the pharmaceutical industry an ‘easy route’ to market exclusivity, without fear of challenges. Indeed, it seems that data exclusivity is meant to increase the (already significant) profitability of the pharmaceutical industry, rather than allowing them to have a legitimate demand fulfilled.

## Notes

1 G. Perry. Data Exclusivity—A Major Threat to Access to Affordable Medicines. *Business Briefing: Pharmagenerics* 2002: 16.

2 By ‘pharmaceutical industry’, we refer to originators who develop and market new drugs.

3 For an overview of US and EU policies of including data exclusivity in bilateral and regional trade agreements, see Sections 2.4 and 3, infra.

4 21 U.S.C. Sect. 355(c)(3)(E)(ii) (1984).

5 Directive 2004/27/EC, OJ L No. 136/34‐57: 39.

6 The Canadian Federal Court, in *Bayer v Canada,* suggesting that market exclusivity is properly the province of patent law, has indeed argued that the regulatory authorities do not refer to or use the originator's data and that indirect reliance should not be precluded. See Bayer v. Canada [1999] 1 FC 553‐582, affirmed 87 CPR (3d) 293.

7 Paris Convention on the Protection of Industrial Property (1883), Art. 10bis.

8 See Section 3, infra; The US has even initiated WTO proceedings against Argentina, claiming that Argentina's lack of data exclusivity legislation constituted a violation of Art. 39(3) TRIPS. See UNCTAD‐ICTSD. 2005. *Resource Book on TRIPS and Development*. New York: Cambridge University Press: 532.

9 See Section 3, infra.

10 Patent linkage makes the market approval of a generic drug conditional on the absence of a patent. Before granting marketing authorization, regulatory authorities must check for relevant patents.

11 All US trade agreements referred to in this article are available at: https://ustr.gov/trade-agreements. [Accessed 7 Dec 2015].

12 See, for example, US‐Australia FTA (2005; Art. 17(10)), US‐Morocco FTA (2006; Art. 15(10)), US‐Bahrein FTA (2006; Art. 14(9)), US‐Oman FTA (2009; Art. 15(9)) and US‐South Korea FTA (2007; Art. 18(9)).

13 C.M. Correa. 2010. Data Exclusivity for Pharmaceuticals: TRIPS Standards and Industry's Demands in Free Trade Agreements. In *Research Handbook on the Protection of Intellectual Property under WTO Rules*. C.M. Correa, ed. Cheltenham: Edward Elgar: 713–727.

14 Ibid.

15 C.M. Correa. 2014. The Impact of the Economic Partnership Agreements on WTO Law. In *EU Bilateral Trade Agreements and Intellectual Property: For Better or Worse?* J. Drexl, H.G. Ruse‐Khan and S. Nadde‐Phlix, eds. Heidelberg: Springer: 87–108.

16 All EU trade agreements referred to in this article are available at: http://ec.europa.eu/trade/policy/countries-and-regions/agreements/. [Accessed 7 Dec 2015].

17 WikiLeaks. 2015. TPP Treaty: Intellectual Property Rights Chapter, Consolidated Text (October 5, 2015).

18 The TPP, negotiated between the US, Mexico, Canada, Australia, Malaysia, Chile, Singapore, Peru, Vietnam, New Zealand, Brunei and Japan, came to a successful conclusion on October 5 2015.

19 S.K. Sell. 1998. *Power and Ideas*. Albany: State University of New York Press.

20 P.M. Ryan. 1998. *Knowledge Diplomacy. Global Competition and the Politics of Intellectual Property*. Washington, DC: Brookings Institution Press; P. Drahos & J. Braithwaite. 2002. *Information Feudalism: Who Owns the Knowledge Economy?* London: Earthscan Publications.

21 D. Acquah. Extending the Limits of Protection of Pharmaceutical Patents and Data Outside the EU ‐ Is There a Need to Rebalance? *IIC ‐ International Review of Intellectual Property and Competition Law* 2014; 45: 256–286; S. Adamini et al. Policy Making on Data Exclusivity in the European Union: From Industrial Insterests to Legal Realities. *Journal of Health Politics, Policy and Law* 2009; 34: 980–1006.

22 Adamini et al., *op. cit*. note 21.

23 C.M. Ho. 2011. *Access to Medicine in the Global Economy: International Agreements on Patents and Related Rights*. Oxford: Oxford University Press.

24 International Federation of Pharmaceutical Manufacturers & Associations (IFPMA). 2000. *Encouragement of New Clinical Drug Development: The Role of Data Exclusivity*. Available at: http://www.ifpma.org/fileadmin/content/Publication/IFPMA_2011_Data_Exclusivity__En_Web.pdf: 11. [Accessed 7 Dec 2015].

25 See Section 2.3, supra.

26 Office of the United States Trade Representative (USTR). 2003. *Special 301 Report*. USTR. Available at: https://ustr.gov/archive/Document_Library/Reports_Publications/2003/2003_Special_301_Report/Section_Index.html: 4‐5. [Accessed 7 Dec 2015].

27 In most policy documents addressing the USTR, business interest groups emphasize data exclusivity as an important concern. See, for example, Biotechnology Industry Organization (BIO). 2011. *Biotechnology Industry Organization Special 301 Submission*. Available at: https://www.bio.org/sites/default/files/BIO_2011_Special_301_Submission.pdf: 4; Pharmaceutical Research and Manufacturers of America (PhRMA). 2014. *Pharmaceutical Research and Manufacturers of America Special 301 Submission*. Available at: http://www.phrma.org/sites/default/files/pdf/2014-special-301-submission.pdf: 7; US Chamber of Commerce. 2015. *U.S. Chamber Of Commerce's Global Intellectual Property Center 2015 Special 301 Submission*. Available at: http://www.theglobalipcenter.com/wp-content/uploads/2013/01/Special-301-Submission-GIPC-FINAL.pdf: 13. [Accessed 7 Dec 2015].

28 Pharmaceutical Research and Manufactureres of America (PhRMA). 2013. *Pharmaceutical Research and Manufactureres of America Special 301 Submission*. Available at: http://www.phrma.org/sites/default/files/pdf/PhRMA%20Special%20301%20Submission%202013.pdf: 18. [Accessed 7 Dec 2015].

29 K. Moberg. Private Industry's Impact on U.S. Trade Law and International Intellectual Property Law: A Study of Post‐TRIPS U.S. Bilateral Agreements and the Capture of the USTR. *Journal of the Patent and Trademark Office Society* 2014; 96: 228–256: 246.

30 A full list of the members of ITAC 15 is available at: http://ita.doc.gov/itac/committees/itac15.asp. [Accessed 7 Dec 2015].

31 Moberg, *op. cit*. note 29.

32 Although this is currently being challenged in courts, see I.P. Watch. 2015. *Court Orders USTR To Justify Industry Advisor Confidentiality in TPP*. Intellectual Property Watch. Available at: http://www.ip-watch.org/2015/10/29/ustr-called-on-to-justify-industry-advisor-confidentiality-in-tpp/.[Accessed 7 Dec 2015].

33 Office of the United States Trade Representative (USTR). 2015. *Special 301 Report*. USTR. Available at: https://ustr.gov/sites/default/files/2015-Special-301-Report-FINAL.pdf [Accessed 7 Dec 2015]; Similarly, also the European Commission report on the protection and enforcement of intellectual property rights in third countries explicitly mentions improving ‘data exclusivity’ protection in several countries as policy objective. See European Commission. 2015. *Commission Staff Working Document – Report on the protection and enforcement of intellectual property rights in third countries (SWD (2015) 132 final)*. Available at: http://ec.europa.eu/trade/policy/accessing-markets/intellectual-property/enforcement/index_en.htm. [Accessed 7 Dec 2015].

34 See, for example, Oxfam America. 2011. *Special 301 Review Hearing Statement*. Available at: http://www.regulations.gov/#!documentDetail;D=USTR-2010-0037-0039: 7‐8; Office of the United States Trade Representative (USTR). 2015. *Special 301 Review Public Hearing February 24, 2015, transcript*. Available at: https://ustr.gov/sites/default/files/2015%20Special%20301%20Hearing%20Schedule_0.pdf: 99‐102; Public Citizen. 2015. *Hearing Testimony for the 2015 Special 301 Review, Statement of Peter Maybarduk, Global Access to Medicines Program Director*. Available at: http://keionline.org/sites/default/files/Public_Citizen_2015_Special_301_Review_Hearing_Testimony.pdf: 3. [Accessed 7 Dec 2015].

35 International Federation of Pharmaceutical Manufacturers & Associations (IFPMA). 2011. *Data Exclusivity: Encouraging Development of New Medicines*. Available at: http://www.ifpma.org/fileadmin/content/Publication/IFPMA_2011_Data_Exclusivity__En_Web.pdf: 5. [Accessed 7 Dec 2015].

36 A. Taubman. Unfair Competition and the Financing of Public‐Knowledge Goods: the Problem of Test Data Protection. *Journal of Intellectual Property Law & Practice* 2008; 3: 591–606.

37 IFPMA, *op. cit*. 35, note p. 5.

38 Pharmaceutical Research and Manufactureres of America (PhRMA). 2014. *Pharmaceutical Research and Manufactureres of America Special 301 Submission*. Available at: http://www.phrma.org/sites/default/files/pdf/2014-special-301-submission.pdf: 10. [Accessed 7 Dec 2015].

39 Biotechnology Industry Organization (BIO). 2013. *The Trans‐Pacific Partnership and Innovation in the Bioeconomy: The Need for 12 Years of Data Protection for Biologics*. Available at: https://www.bio.org/articles/trans-pacific-partnership-and-innovation-bioeconomy-need-12-years-data-protection-biologi-0. [Accessed 7 Dec 2015].

40 Taubman, *op. cit*. note 36, p. 593.

41 See for example PhRMA, *op. cit*. note 28, pp. 11, 89, 106; IFPMA, *op. cit*. note 35, p. 6.

42 See for example Pharmaceutical Research and Manufactureres of America (PhRMA). 2013. *Statement of Jeffrey K. Francer Vice President and Senior Counsel Pharmaceutical Research and Manufacturers of America Before the Committee on Strategies for Responsible Sharing of Clinical Trial Data (Institute of Medicine National Academy of Sciences, October 23, 2013)*. PhRMA. Available at: http://phrma.org/sites/default/files/pdf/PhRMA-Data-Sharing-Testimony-10-23-13-final.pdf: 5; GlaxoSmithKlein (GSK). 2014. *GSK Public policy positions: Regulatory Data Protection* GlaxoSmithKline Communications and Government Affairs. Available at: https://www.gsk.com/media/280896/regulatory-data-protection-policy.pdf: 3. [Accessed 7 Dec 2015].

43 Taubman, *op. cit*. note 36.

44 Regulation 536/2014/EU, OJ L No. 158/1‐76, mandates that, when clinical trials are conducted for the purpose of regulatory approval, the clinical study reports (which accompany the application for regulatory approval, see art. 2 (2) (35)) need to be submitted to the EU database, within 30 days after the final marketing authorization decision. (Art. 37(4)) Art. 81 explicitly provides that the database shall be publicly accessible. See also European Medicines Agency (EMA). 2014. *European Medicines Agency policy on publication of clinical data for medicinal products for human use of 2 October 2014 (EMA/240810/2013)*. Available at: http://www.ema.europa.eu/docs/en_GB/document_library/Other/2014/10/WC500174796.pdf. [Accessed 7 Dec 2015].

45 National Institutes of Health (NIH). 2014. *HHS and NIH take steps to enhance transparency of clinical trial results*. Available at: http://www.nih.gov/news/health/nov2014/od-19.htm. [Accessed 7 Dec 2015].

46 S. Morgan et al. The Cost of Drug Development: A Systematic Review. *Health Policy* 2011; 100: 4–17.

47 In an effort to propagate an anti‐drug‐regulation position, the CSDD was established as a vehicle to legitimize industry's claims regarding the ‘adverse’ effects of government interference and to avoid the US government's insistence on lower drug prices. While affiliated with the University of Rochester and later Tufts, its funding came directly from industry. See E. Nik‐Khah. Neoliberal pharmaceutical science and the Chicago School of Economics. *Social Studies of Science* 2014: 1–29.

48 Tufts Center for the Study of Drug Development (CSDD). 2014. *Cost to Develop and Win Marketing Approval for a New Drug Is $2.6 Billion*. Available at: http://csdd.tufts.edu/news/complete_story/pr_tufts_csdd_2014_cost_study. [Accessed 7 Dec 2015].

49 D.W. Light & J.R. Lexchin. Pharmaceutical Research and Development: What Do We Get for All That Money? *BMJ* 2012; 345: 1‐5.

50 Pharmaceutical Research and Manufactureres of America (PhRMA). 2011. *2011 Profile: Pharmaceutical Industry*. Available at: http://www.phrma-jp.org/archives/pdf/profile/PhRMA%20Profile%202011%20FINAL.pdf. [Accessed 7 Dec 2015].

51 Drug.com. 2013. *U.S. Pharmaceutical Sales – 2013*. Available at: http://www.drugs.com/stats/top100/2013/sales. [Accessed 7 Dec 2015].

52 EvaluatePharma. 2014. *World Preview 2014, Outlook to 2020*. Available at: http://info.evaluategroup.com/rs/evaluatepharmaltd/images/EP240614.pdf. [Accessed 7 Dec 2015].

53 K. Maskus. The New Globalisation of Intellectual Property Rights: What's New This Time? *Autralian Economic History Review* 2014; 54: 262‐284.

54 J. Lerner. The Empirical Impact of Intellectual Property Rights on Innovation: Puzzles and Clues. *The American Economic Review* 2009; 99: 343‐348.

55 Y. Qian. Do National Patent Laws Stimulate Domestic Innovation in a Global Patenting Environment? A Cross‐Country Analysis of Pharmaceutical Patent Protection, 1978–2002. *The Review of Economics and Statistics* 2007; 89: 436‐453; J. Hudson & A. Minea. Innovation, Intellectual Property Rights, and Economic Development: A Unified Empirical Investigation. *World Development* 2013; 46: 66‐78.

56 Maskus, *op. cit*. note 53; B.B. Allred & W.G. Park. Patent Rights and Innovative Activity: Evidence from National and Firm‐level Data. *Journal of International Business Studies* 2007; 38: 878‐900. Y. Chen & T. Puttitanun. Intellectual property rights and innovation in developing countries*. Journal of Development Economics* 2005; 78: 474‐493.

57 Lerner, *op. cit*. note 54.

58 Adamini et al., *op. cit*. note 21.

59 Maskus, *op. cit*. note 53.

60 Ibid.

61 Allred & Park, *op. cit*. note 56.

62 Oxfam International. 2007. Oxfam Briefing Paper: All costs, no benefits: How TRIPS‐plus intellectual property rules in the US‐Jordan FTA affect access to medicines. Available at: https://www.oxfam.org/sites/www.oxfam.org/files/all%20costs,%20no%20benefits.pdf: 15‐17. [Accessed 7 Dec 2015].

63 Maskus, *op. cit*. note 53.

64 See also K. Maskus. 2012. *Private Rights and Public Problems: The Global Economics of Intellectual Property in the 21th Century*. Washington, DC: Peterson Institute for International Economics: 35‐64.

65 See Y. Qian. Do National Patent Laws Stimulate Domestic Innovation in a Global Patenting Environment? A Cross‐Country Analysis of Pharmaceutical Patent Protection, 1978–2002. *The Review of Economics and Statistics* 2007; 89: 436‐453.

66 Allred & Park, *op. cit*. note 56; Chen & Puttitanun, *op. cit*. note 56.

67 M.K. Kyle & A.M. McGahan. Investments in Pharmaceuticals Before and After TRIPS. *The Review of Economics and Statistics* 2012; 94: 1157‐1172.

68 L.D. Qiu & H. Yu. Does the Protection of Foreign Intellectual Property Rights Stimulate Innovation in the US? *Review of International Economics* 2010; 18: 882‐895: 883.

69 Adamini et al., *op. cit*. note 21.

70 United Nations (UN). 2012. *Millennium Development Goal 8 ‐ The Global Partnership for Development: Making Rhetoric a Reality ‐ MDG Gap Task Force Report 2012*. Available at: http://www.un.org/millenniumgoals/2012_Gap_Report/MDG_2012Gap_Task_Force_report.pdf. [Accessed 7 Dec 2015].

71 UN, *op. cit*. note 70, pp. 66‐70; World Health Organization (WHO). 2011. *The World Medicines Situation 2011 ‐ Medicines Prices, Availability and Affordability (3rd Edition)*. Available at: http://www.who.int/medicines/areas/policy/world_medicines_situation/WMS_ch6_wPricing_v6.pdf: 13‐14. [Accessed 7 Dec 2015].

72 E.R. Shaffer & J.E. Brenner. A Trade Agreement's Impact on Access to Generic Drugs. *Health Affairs* 2009; 28: 957‐968.

73 Ibid.

74 WHO, *op. cit*. note 71, pp. 5‐6.

75 Other mechanisms such as tiered pricing – i.e. differentiated pricing based on the customers’ financial means – can also lower the price of medicines. However, empirical data show that generic competition remains the most effective in in achieving the lowest sustainable prices. As long as a drug can be sold exclusively, an incentive remains to charge the highest possible price in that market. See S. Moon et al. A win‐win solution?: A critical analysis of tiered pricing to improve access to medicines in developing countries. *Globalization and Health* 2011; 7: 39.

76 Compulsory licensing authorizes the use of a patented product or process without permission of the patent holder.

